# Considerations for the Use of Photobiomodulation in the Treatment of Retinal Diseases

**DOI:** 10.3390/biom12121811

**Published:** 2022-12-03

**Authors:** Chun-Xia Zhang, Yan Lou, Jing Chi, Xiao-Li Bao, Bin Fan, Guang-Yu Li

**Affiliations:** 1 Department of Ophthalmology, The Second Hospital of Jilin University, Changchun 130042, China; 2 Department of Nephropathy, The Second Hospital of Jilin University, Changchun 130042, China

**Keywords:** PBM, retinal diseases, red light, light irradiation, illumination parameters

## Abstract

Photobiomodulation (PBM) refers to the beneficial effect produced from low-energy light irradiation on target cells or tissues. Increasing evidence in the literature suggests that PBM plays a positive role in the treatment of retinal diseases. However, there is great variation in the light sources and illumination parameters used in different studies, resulting in significantly different conclusions regarding PBM’s therapeutic effects. In addition, the mechanism by which PBM improves retinal function has not been fully elucidated. In this study, we conducted a narrative review of the published literature on PBM for treating retinal diseases and summarized the key illumination parameters used in PBM. Furthermore, we explored the potential molecular mechanisms of PBM at the retinal cellular level with the goal of providing evidence for the improved utilization of PBM in the treatment of retinal diseases.

## 1. Introduction

Photobiomodulation (PBM) refers to the beneficial effect of low-energy light irradiation on target cells or tissues [1]. Recently, numerous studies have shown that PBM also plays a therapeutic role in various retinal diseases. Merry et al. found that the application of PBM (670 nm, 4–7.7 J/cm^2^, 590 nm and 790 nm 0.1 J/cm^2^, 3 weeks) significantly increased the best corrected visual acuity (BCVA) and contrast sensitivity (CS) in patients with dry age-related macular degeneration (AMD) and reduced the drusen volume, indicating that PBM produced from low-energy red light exposure may alleviate the progression of dry AMD and improve the visual function of patients [2]. In addition, Shen et al. showed that exposure to red laser light (670 nm; 18 J/cm^2^, 5 weeks) significantly improved diabetic macular edema (DME) and reduced the foveal thickness [3]. Natoli et al. demonstrated that the irradiation of red narrow-band light-emitting diodes (LEDs) (670 nm, 9 J/cm^2^) ameliorated retinal microvascular occlusion and suppressed neovascularization in hyperoxia-induced retinopathy in rats [4]. Interestingly, Eells et al. showed that the exposure to red LED light (670 nm, 4 J/cm^2^) also alleviated methanol-induced toxicity in rat retinas [5]. In recent years, Zhu et al. reported the mechanism of action of PBM and its therapeutic effects on several retinal diseases in detail [6,7,8,9]. However, the parameters of PBM that play a role in different retinal diseases were not analyzed and summarized in detail, and determining the type of light suitable for the parameter conditions is key to understanding the role of PBM. Therefore, unlike previous reviews, this study mainly reviews the radiation parameters of PBM and examines the most potentially effective wavelength and energy range of PBM in the treatment of retinal diseases. In addition, we explored the potential molecular mechanism of PBM at the retinal cell level and summarized the current situation of PBM in the treatment of retinal diseases in recent years to provide evidence to help improve the application of PBM in the treatment of retinal diseases.

## 2. Methods

The PubMed database and Web of Science were searched using multiple combinations of the keywords “PBM”, “red light”, “photobiomodulation”, “retinal diseases“, and “retinal”. Articles published up to 2022 were retrieved, and articles without an available English translation were excluded.

## 3. Wavelength of Light for Exerting PBM

The cornea of the human eye can filter out light wavelengths below 295 nm, while the lens crystal can remove light wavelengths in the range of 300–400 nm. Therefore, the light wavelengths that reaches the retina are usually greater than 400 nm [10]. Red light has a longer wavelength and lower frequency in the visible light spectrum, carrying less energy than short wavelength light such as blue or purple light [11]. According to the action spectrum, which is a curve that illustrates the relationship between the different light wavelengths and sensitivity caused by retinal photochemical damage, the energy required for red light to induce retinal photochemical damage is far higher than that required by other short wavelength lights. Therefore, the risk of red light-induced retinal photochemical damage is lowest when irradiating within the energy range of common applications [12]. Red light and near-infrared light (wavelength range of 600–1000 nm) can reach the retina through the refractive system, which may play a major role in triggering PBM. For example, Shen et al. found that irradiation with a 670 nm red laser (18 J/cm^2^, 5 weeks) significantly reduced the central macular thickness (CMT) in diabetic macular edema and improved visual function [3]. Heinig et al. showed that exposure to red light (670 nm, 32. 4 J/cm^2^) and near-infrared LED light (810 nm, 32. 4 J/cm^2^) enhanced the mitochondrial energy metabolism of retinal neurons and increased the production of ATP, thereby ameliorating blue light-induced damage in mice photoreceptors [13]. Cheng et al. observed that irradiation with red light (670 nm, 5 J/cm^2^, 8 months) significantly inhibited the leakage and degeneration of retinal capillaries in diabetic mice and improved visual function [14].

Cytochrome C oxidase is regarded as the dominant molecular group in cells and it absorbs photon energy and triggers the effect of PBM. Karu et al. demonstrated that the spectrum region of 620 nm and 710–790 nm matches the absorption peak of reduced cytochrome C oxidase, while the spectrum region of 650–680 nm and 820–830 nm fits the absorption peak of oxidized cytochrome C oxidase [15,16,17]. Wong-Riley et al. also found that among the wavelengths of 670 nm, 728 nm, 770 nm, 830 nm, and 880 nm, the 830 nm and 670 nm wavelengths were consistent with the absorption spectrum of oxidized cytochrome C oxidase. Furthermore, light with these wavelengths exhibited the best effect on increasing neuronal energy metabolism, while light with a 728 nm wavelength had the least beneficial effect on triggering PBM [18]. In addition, Gupta et al. observed that irradiation with light with 635 and 810 nm wavelengths significantly promoted the healing process of dermal abrasions in mice, while 730 and 980 nm lights failed to show positive effects on healing [19].

Due to the special structure of the eye, the selection of effective wavelengths of light is key to the application of PBM for the treatment of retinal diseases. Red to near-infrared light (600–1000 nm) can reach the retina through the eye’s refractive system with less damage to cells or tissues, and this is the wavelength range of choice for treating retinal diseases. Red to near-infrared light photons with long wavelengths can directly transfer energy to cytochrome C oxidase, leading to an increase in enzyme activity and energy metabolism, which may play a key role in further inducing PBM. Based on the literature summary above, light wavelengths at 635–680 nm and 810–830 nm are more suitable for inducing PBM to treat retinal diseases.

## 4. Energy Density of Light Irradiation

For retinal treatments, Tang et al. found that culture with high concentrations of glucose (30 mM) for 4 days led to the death of 661 W photoreceptor-like cells, human retinal pigmental epithelial (ARPE19) cells, retinal ganglion cells (RGCs), and Muller cells. Irradiation (25 mW/cm^2^) with red light (670 nm) for 50 s failed to attenuate high glucose-induced 661 w cell death, while the same irradiation for 200 s was able to significantly reduce 661 w cell death. Moreover, irradiation with 670 nm red light (25 mW/cm^2^) for 200 s protected both RGC and Muller cells from high-glucose toxicity, but had no effect on ARPE19 cells. In addition, Tang et al. also verified that the use of 670 nm red light (25 mW/cm^2^) for 4 min significantly mitigated the death of retinal ganglion cells in diabetic mice in vivo [20]. Shen et al. observed that irradiation with 670 nm red light (200 mW/cm^2^ 90 s, 5 weeks) significantly reduced the CMT in patients with DME 2 months after treatment. However, irradiation with 670 nm red light (25 mW/cm^2^, 100 mW/cm^2^, 90 s, 5 weeks) failed to attenuate CMT in patients with DME [3], indicating that there is also a bidirectional dose–curved relationship between the irradiation energy and the biological effect of PBM for the retina [3,20] and different retinal cells may require different irradiation energies to exert PBM effects.

The retina is composed of various neurons and glial cells, and the structural and functional integrity of the retina depends on various cellular interactions. Different retinal cells may require different irradiation energies to exert PBM effects. Therefore, the selection of safe and effective irradiation energy (mainly irradiance and energy density) is a challenge for PBM treatment of retinal diseases. We suggest that the energy response curves of retinal cells under different retinal disease models could be established to provide a reference for the selection of irradiation energy for the PBM treatment of retinal diseases. An irradiance of less than 50 J/cm^2^ is suggested [21].

## 5. Parameters of Light Irradiation

Due to the differences observed in the light source and irradiation parameters (wavelength, energy, duration, protocol) of PBM for treating retinal diseases, we have summarized the animal and clinical studies found in the recent literature (2012–2022) in Table 1, Table 2 and Table 3.

In animal models of retinal diseases, the 670 nm LED is the most commonly used light source, and direct irradiation on the eye or diffuse irradiation of ambient light is the most investigated approach. As for the parameters of light irradiation, the energy density range is normally between 4 and 9 J/cm^2^, the irradiance is between 20 and 60 mW/cm^2^, and the irradiation duration is from 80 to 240 s (Table 1).

Some clinical studies have shown that PBM plays a positive role in the treatment of dry AMD and diabetic macular edema, but the parameters for the light irradiation vary greatly. Monochromatic red light (670 nm) or a combination of multiple wavelength lights is most often adopted as the light source in clinics. Direct irradiation of the affected eye is also commonly used, with the eyelid closed and/or open, wherein the irradiation energy for the closed eyes is often higher than that for the open eye. The parameters of irradiation used have varied widely, with the variation in the energy density ranging from 0.1 to 25 J/cm^2^, the variation in the irradiance ranging between 8 and 200 mW/cm^2^, and an irradiation duration anywhere between 80 and 250 s (Table 2). However, several studies have reported that PBM plays a negative role in the treatment of progressed AMD, especially accompanied by DME with good visual eyesight [32,33,34] (Table 3). Therefore, the progressive stage of retinal diseases is another factor that should be considered when adopting PBM to achieve the best clinical effects.

## 6. Potential Molecular Mechanisms of PBM for Treating Retinal Diseases

### 6.1. Acting on Cytochrome C Oxidase to Increase Energy Supply

In the late 1980s and 1990s, Passarella et al. discovered that irradiation with near-infrared light significantly increased the synthesis of adenosine triphosphate (ATP) from the isolated mitochondria of rat liver and elevated the level of O_2_ consumption, which are indicative of improved mitochondrial function after near-infrared light irradiation [35]. Subsequently, Karu et al. further verified that cytochrome C oxidase is the major photosensitive group absorbing the photon energy of visible light and near-infrared light [36]. After the energy of a photon transfers to cytochrome C oxidase, it can alter the redox status and increase the rate of electron transfer, thereby promoting the production of ATP [37,38]. Formic acid, a toxic metabolite of methanol, can inhibit cytochrome C oxidase activity. Eells et al. showed that red light exposure (670 nm LED, 4 J/cm^2^) significantly protects the retina from formic acid-induced damage, which further indicates that cytochrome C oxidase plays a major role in absorbing photon energy in cells [5].

### 6.2. Influencing Intracellular Redox Levels

**Figure 1:**
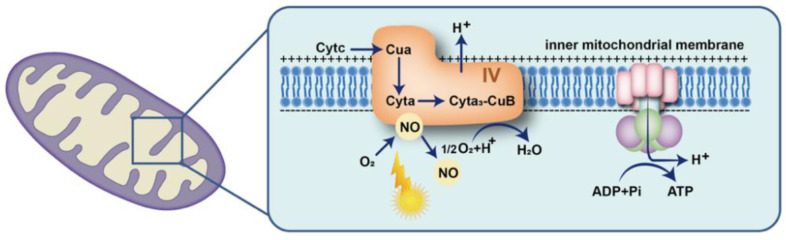
Schematic representation of the influence of light on intracellular redox levels. Photon energy facilitates the dissociation of nitric oxide (NO) from the binding site of cytochrome C oxidase and subsequently promotes oxygen binding to the dissociated site, to enhance mitochondrial respiration and ATP generation.

In the 1990s, Moncada and Erusalimsky et al. first demonstrated that nitric oxide (NO) can bind to cytochrome C oxidase and inhibit the mitochondrial respiratory chain of mammalian cells [39]. However, many studies have demonstrated that irradiation with red light may significantly increase the level of intracellular NO [1,40,41]. The transfer of photon energy may facilitate the dissociation of NO from the binding site of the heme iron and copper centers of cytochrome C oxidase, and subsequently promote oxygen binding to the dissociated site to enhance mitochondrial respiration and reactive oxygen species (ROS) and ATP generation (Figure 1) [41]. Furthermore, the increased ROS may function as a second messenger to regulate various signaling pathways affecting gene expression [42]. However, Kim et al. found that PBM pretreatment inhibited ROS production in a hypoxia model of retinal pigmental cells (RPE) [43]. Huang et al. also demonstrated that low-level laser treatment (LLLT) increased ROS in normal neurons but decreased ROS in oxidatively stressed neurons, suggesting that this might be related to the fact that low-level laser can increase mitochondrial membrane potential (MMP). LLLT leads to an increase in MMP in normal cells, with a consequent increase in ROS levels, while MMP decreases in oxidatively stressed cells. When LLLT is delivered to cells, MMP increases, and thus the amount of ROS produced by mitochondria decreases [44].

### 6.3. Regulating Cellular Inflammatory Factor Release

Irradiation with red light not only increases the rate of ATP production in mitochondria, but it can also suppress the inflammatory response [20,24,45]. Kokkinopoulos et al. found that irradiation with red light (670 nm, 3.6 J/cm^2^, 5 times) significantly suppressed retinal inflammation in aged mice. After red light irradiation, the number of macrophages in the retina were markedly decreased, and the levels of the pro-inflammatory factor tumor necrosis factor α (TNF-α), the inflammatory marker complement component 3d (C3d), and calcitonin were also significantly reduced [45]. Similarly, Lu et al. showed that irradiation with red light (670 nm, 9 J/cm^2^, 5 days) can substantially inhibit the activation, proliferation, and migration of retinal Müller cells, suppress retinal inflammation in rats induced by visible light damage, significantly mitigate the formation of glial scars, and improve the survival of photoreceptors [24]. In addition, Tang et al. also demonstrated that irradiation with red light (670 nm, 6 J/cm^2^) significantly attenuated leukostasis and reduced the expression of intercellular adhesion molecule 1 (ICAM-1) in diabetic mice retina [20].

Red and near-infrared light irradiation can dissociate NO from cytochrome C oxidase and subsequently increase the contact of oxygen with cytochrome C oxidase, which in turn promotes mitochondrial respiration rate, increases ATP synthesis, and alters intracellular oxidation levels. In addition, red light irradiation can modulate the release of cellular inflammatory factors, thereby suppressing inflammation. Therefore, PBM may have a therapeutic effect on retinal diseases associated with energy abnormalities and inflammation.

## 7. Biological Effect of PBM on Retinal Cells

The eye is one of the most complex sensory organs of the human body [46]. The retina is the extension of the central nervous system (CNS) at the back of the eye, which is responsible for the formation of vision. The neural tissue is responsible for detecting and processing the initial vision, including brightness, contrast, direction, and speed, and then sending these to other areas of the CNS through the optic nerve for further processing [47]. Similar to all mammalian retinas, the human retina is mainly composed of a neuroepithelium and pigment epithelium; the retinal neuroepithelium is in turn composed of neurons and glia, including photoreceptors, bipolar cells, ganglion cells, horizontal cells, and amacrine cells. When photoreceptor cells receive photons, they convert light signals into electrochemical signals and connect them to bipolar interneurons. These cells then contact ganglion cells, which eventually transmit signals to the brain [46]. Müller cells are the most abundant type of glial cells in the retina. They are the only cells that span the entire width of the retina and have close contact with retinal blood vessels and retinal neurons. Because of this arrangement, Müller cells have many important functions in the retina, such as recycling neurotransmitters, supplying nutrients to the retina, and maintaining the blood–retinal barrier [48]. Retinal pigment epithelium is a cubic epithelial cell located between photoreceptors and the Brucella membrane that interacts with photoreceptors to maintain visual function. It carries out processes such as the transport of nutrients and metabolites, phagocytosis of the outer membrane disc of photoreceptors, and synthesis of growth factor [49]. Here, we further discuss the biological effects of PBM on the key retinal cells mentioned above.

Retinal ganglion cells (RGCs) located in the inner layer of the retina are responsible for receiving visual signals from photoreceptors and for transmitting these signals to the visual cortex of the brain through the optic nerve [50]. Accomplishing these physiological functions requires a sufficient ATP supply; therefore, RGCs contain a large number of mitochondria to match the high energy demands. However, once the function of the electron transport chain is compromised, it also causes the generation of intracellular ROS, which may lead to oxidative stress-induced damage in proteins, lipids, and nuclear DNA, thus triggering cell death cascades [51]. PBM may have a direct influence on the electron transport chain of mitochondria in order to increase the rate of ATP production and protect RGCs against damage due to insufficient energy or energy exhaustion. Beirne et al. showed that the dendritic length and area of RGCs in cultured retinal explants decreased significantly 8–16 h after dissection, while irradiation with red light (670 nm, 31.7 J/cm^2^) significantly attenuated the reduction in dendrite length and area in vitro [52]. In addition, Beirne et al. also demonstrated that red light (670 nm, 4 J/cm^2^, 5 days) irradiation significantly mitigated the decline in the dendritic length and area of RGCs in optic nerve crush (ONC) injured mice in vivo, indicating that PBM produced from red light irradiation can alleviate dendritic lesions of RGCs caused by axonal injury [53].

Human photoreceptor cells consist of cones and rods [54]. The inner segment of photoreceptor cells also contains a large number of mitochondria to generate sufficient ATP for energy consumption in visual transduction [55]. Other studies have shown that with aging, the mitochondrial function of photoreceptor cells gradually declines and is accompanied by reduced mitochondrial membrane potential and ATP production but increased generation of ROS [56,57]. By 70 years of age, there is a nearly 30% rod loss compared to younger individuals, and a remarkable decline in cone function related to the progression of various retinal diseases [51]. By measuring the scotopic threshold response and color contrast sensitivity (CCS) to assess the function of cones and rods in individuals aged 28–72 years, Shinhmar et al. found that the value of scotopic threshold response and color contrast sensitivity (CCS) decreased remarkably after the age of 40 years. However, irradiation with red light (670 nm, 7.2 J/cm^2^, 2 weeks) significantly enhanced the activity of mitochondrial enzyme complexes and increased the mitochondrial membrane potential and ATP production in photoreceptor cells, which attenuated the decline in the scotopic threshold response and color contrast sensitivity (CCS) in individuals aged over 40 years [58]. In addition, Gopalakrishnan et al. found that irradiation with near-infrared light (830 nm, 4.5 J/cm^2^, 5 days on, 2 days off) significantly improved the metabolic status of retinal neurons and retinal function and mitigated the death of photoreceptor cells in P23H transgenic rats (a retinitis pigmentosa model) [28]. These studies indicate that PBM may exert a positive effect on attenuating the dysfunction of photoreceptor cells by enhancing energy metabolism and mitochondrial function.

Müller cells, a major type of glial cell, are evenly distributed in the mammalian retina and are crucial for supporting the structure of retinal vascular neurons and for maintaining retinal homeostasis [24]. When the retina is damaged due to severe insults, a secondary inflammatory response is induced, which may cause the proliferation of Müller cells and the formation of retinal glial scars [22]. Lu et al. found that irradiation with red light (670 nm, 9 J/cm^2^, 3 times) markedly inhibited the activation and proliferation of Müller cells in vitro and significantly suppressed the activation, proliferation, and migration of Müller cells and inhibited inflammation in light-damaged rat retinas in vivo [24]. Thus, PBM also plays a positive role in suppressing retinal inflammation and in the activation of Müller cells.

The retinal pigment epithelium (RPE) is located between photoreceptors and the Bruchs’ membrane to form the outer retina–blood barrier, and it has various important physiological functions including visual cycles, transporting nutrients and metabolites, phagocytizing the outer segment membranes of photoreceptors, and synthesizing growth factors [49]. Kokkinopoulos et al. showed that irradiation with red light (670 nm, 3.6 J/cm^2^, 5 times) significantly attenuated mitochondrial membrane polarization and increased ATP production in the RPE cells of aged mice [45]. In addition, Fumar et al. demonstrated that irradiation with red light (670 nm, 2500 lx, twice a day, 250 s/per time, 4 d) significantly improved oxidative stress-induced hypoactivity of phagocytosis in ARPE-19 cells and human primary retinal pigment epithelium (hRPE) cells in vitro and reduced ROS production [59]. These studies suggest that PBM produced by red light irradiation may improve the function of retinal cells in many pathological conditions through promoting energy production and suppressing inflammatory responses.

Retinal neuronal cells (such as RGCs and photoreceptor cells) and retinal pigment epithelial cells contain a large number of mitochondria to ensure that enough ATP is produced to meet the high metabolic demands of the retina. However, when mitochondrial function is impaired, these retinal cells are also more susceptible to oxidative stress-induced damage and secondary inflammation, leading to Müller cell proliferation and retinal glial scar formation. PBM can increase the mitochondrial respiration rate to increase ATP synthesis and inhibit inflammation, and may be an effective treatment for retinal damage caused by abnormal energy metabolism in retinal cells.

## 8. Therapeutic Effect of PBM on Retinal Diseases

Age-related macular degeneration is the leading cause of irreversible vision loss in people over 60 years in developed countries [60,61]. There are two types of AMD, namely, atrophic (dry) and exudative (wet) [62]. The formation of macular drusen below the RPE is a major feature of atrophic AMD. Merry et al. found that irradiation with light of multiple wavelength combinations (670 nm, 4–7.7 J/cm^2^, 590 nm and 790 nm 0.1 J/cm^2^, 3 weeks) could significantly reduce the volume of drusen and improve BCVA and CS in atrophic AMD patients [2]. Similarly, Markowitz and Devenyi et al. also found that irradiation with light of multiple wavelengths (590 nm = 5 mW/cm^2^, 660 nm = 65 mW/cm^2^, 850 nm = 8 mW/cm^2^, a total of 250 s, 3× per week for 3–4 weeks over 1 year) could reduce the volume of drusen and improve the visual function in patients with dry AMD, with repeated therapy suggested to enhance long-term effects [30]. Ultimately, these studies indicate that irradiation with low-energy red light can slow down the progression of retinal diseases and improve visual function.

Diabetic macular edema is a common complication in diabetic retinopathy due to the impaired blood-retinal barrier and increased vascular permeability in the macula, which commonly cause impaired vision [63]. Low-energy red light irradiation may significantly mitigate oxidative-stress induced damage and suppress inflammatory responses in the retina. Shen et al. showed that irradiation with a red laser (670 nm, 18 J/cm^2^, 5 weeks) significantly reduced the foveal thickness in DME patients 2–6 months after treatment [3]. Similarly, Tang and Herda et al. demonstrated that exposure to red light (670 nm, 25 J/cm^2^, once a day for 2–9 months) significantly decreased the central retinal thickness in DME without causing complications [31].

Acute retinal light injury is manifested as the damage or death of photoreceptors and is accompanied by the loss of RPE, gliosis of Müller cells, and occlusion of the choroidal vessels [64]. Albarracin et al. found that irradiation with red LED light (670 nm, 9 J/cm^2^) significantly inhibited the secretion of inflammatory factors, inhibited microglia and macrophage infiltration, and attenuated the death of photoreceptor cells in visible light-damaged rat retinas [64]. Heinig et al. showed that irradiation with red light (670 nm, 32.4 J/cm^2^) and near-infrared light (810 nm, 32.4 J/cm^2^) significantly increased the mitochondrial energy metabolism and ATP production of retinal neurons, thereby mitigating blue light-induced damage in mouse retinas [13]. Further, low-energy red light irradiation may enhance cellular energy metabolism and suppress retinal inflammation, which protects the retina against light damage [64].

Myopia, especially high myopia, is accompanied by a high risk of severe retinal complications, such as retinal detachment, CNV, and retinal degeneration [65,66]. Increased evidence indicates that longer durations of outdoor activity and light exposure can significantly lower the incidence of myopia in adolescents [67,68,69]. Gawne et al. showed that exposure to narrow-band red light (624 or 636 nm, 527–749 lux) significantly inhibited the axial growth in infant tree shrews and produced hyperopia, which caused shortened vitreous cavities and increased choroid thickness compared to those infants reared under natural light, and hyperopia produced by the red light irradiation even lasted until the juvenile stage or adolescence [70,71]. Furthermore, Hung et al. observed that exposure to red light greatly reduced the likelihood of infant monkeys developing FDM/compensating myopia in response to imposed hyperopic defocus [72]. In addition, red-light-induced alterations in refractive development increased choroidal thickness and were associated with reduced vitreous chamber elongation. More recently, a clinical study conducted by Jiang et al. showed that after 12 months of exposure to 650 nm red light (0.29 mw, 3 min, twice daily, 5 days per week), the axial growth and refraction alternation in myopic children aged 8–13 years were significantly suppressed as compared with those in the non-irradiated group, indicating that repeated low-level red light irradiation plays a positive role in controlling myopia progression [73]. Gawne et al. also proposed a hypothesis that the emmetropization mechanisms in humans decrease with age; however, red light irradiation might reactivate emmetrope signals to prevent and control myopia [71].

AMD, DME, and retinal light injury are common diseases of the eye, and retinal damage mainly occurs in retinal photoreceptors, Müller cells, and retinal pigment epithelial cells. In this section, we listed the therapeutic effects of PBM on retinal diseases, and found that PBM’s most likely mechanism of treatment is that it can enhance the mitochondrial respiration rate and inhibit inflammation. In addition, the animal and clinical literature has reported a positive effect of red light in the prevention and control of myopia. Although the mechanism by which low-energy red light irradiation controls myopia development has not been fully elucidated, this study provides a new direction and strategy for myopia prevention and control.

## 9. Summary

The high metabolic features of the retina require high energy supply, and therefore, most retinal neurons contain a large number of mitochondria to produce sufficient ATP. However, when the mitochondrial function is compromised, it leads to oxidative-stress induced damage in the retina. Light irradiation via PBM can increase the rate of mitochondrial ATP production and suppress inflammatory responses in the retina, thus playing a positive therapeutic role in many retinal diseases. Red to near-infrared light wavelengths in the range of 635–680 nm and 810–830 nm are suitable for inducing PBM to treat retinal diseases, while a 670 nm red laser or LED light wavelength is the most reported light source wavelength in the literature for inducing PBM to treat retinal diseases. Irradiance and energy density are the two key parameters that need to be carefully controlled during PBM therapy. The stage of retinal disease progression is another factor that should be considered when adopting PBM to achieve the best clinical effects.

## Figures and Tables

**Table 1:** Animal studies on PBM for the treatment of retinal diseases.

Type of Retinal Disease	First Author (Year)	Subject (No.)	λ (nm)	Dose	Irradiance (Duration)	Illumination Method	Result
Retinal Degeneration	Albarracin 2012 [22]	rats (36)	670 nm LED	9 J/cm^2^	60 mW/cm^2^ 3 min 5 days	Eye level was approximately 2.5 cm away from the light source	Ameliorates the light-induced alterations in the expression of Müller-cell specific markers for structure, stress, metabolism, and inflammation
Retinal Degeneration	Marco 2013 [23]	rats	670 nm LED	5 J/cm^2^	3 min 2–10 days	The WARP 75’s emission plate was placed on the “ceiling” 1–2 cm from the animal’s eye	Reduces photoreceptor death, preserves the population of surviving photoreceptors, and reduces the upregulation of glial fibrillary acidic protein in Müller cells
Retinal Degeneration	Lu 2018 [24]	rats (39)	670 nm LED	9 J/cm^2^	60 mW/cm^2^, 3 min 5 days	Animals were positioned so that both eyes were approximately 2.5 cm away from the light source	Mitigates the production of Müller cell-related pro-inflammatory cytokines, reduces microglia/macrophage (MG/MΦ) recruitment into the outer retina
Retinal Degeneration	Di Paolo 2021 [25]	rats (25)	670 nm LED	4.0–4.5 J/cm^2^	3 min 7 days	Placed 2.5 cm away from the animal	Preserves retinal thickness and gliosis and microglia invasion
DR	Tang 2013 [20]	rats (80)	670 nm LED	6 J/cm^2^	25 mW/cm^2^ 240 s 10 weeks	Approximately 1 inch above the animal, and exposed to whole-body irradiation	Ameliorates lesions of DR in vivo
DR	Saliba 2015 [26]	C57BL/6J mice (60)	670 nm LED	4.8 J/cm^2^	20 mW/cm^2^ 240 s 10 weeks	2–3 cm distance used between the device and the animal	Inhibits early changes of DR
DR	Cheng 2018 [14]	C57BL/6J mice	670 nm LED	5 J/cm^2^	25 mW/cm^2^ 240 s 8 months	The light provided illumination evenly across the entire back of the animals.	Inhibits the functional and histopathologic features of early DR
ROP	Natoli 2013 [4]	C57BL/6J mice or rats	670 nm LED	9 J/cm^2^	50 mw/cm^2^ 3 min (P7–P17, mice) (P0–P18, rats)	Each animal was held approximately 2.5 cm from the light source	Neovascularization, vaso-obliteration, and abnormal peripheral branching patterns of retinal vessels in oxygen-induced retinopathy
AMD	Begum 2013 [27]	mice (29)	670 nm LED	7.2 J/cm^2^	20 mW/cm^2^ 6 min twice a day 14 days	In the form of supplemented environmental light	Reduces inflammation probably via cytochrome c oxidase activation in mice even
RP	Gopalakrishnan 2020 [28]	rats	830 nm LED	4.5 J/cm^2^	25 mW/cm^2^ 180 s, 5 days per week (p10–p25)	LED array was positioned directly over to the animal’s head at a distance of 2 cm exposing both eyes.	Preserves mitochondrial metabolic state and attenuates photoreceptor loss

PBM device: Quantum Devices, Barneveld, WI. PBM = photobiomodulation, LED = light-emitting diodes, DR = diabetic retinopathy, ROP = retinopathy of prematurity, AMD = age-related macular degeneration, DME = diabetic macular edema, RP = retinitis pigmentosa.

**Table 2:** Clinical studies on PBM for the treatment of retinal diseases.

Type of Retinal Disease	First Author (Year)	Subject (No.)	λ (nm)	Dose	Irradiance Treatment Duration	Illumination Method	Result
AMD	Siqueira 2021 [29]	Human (10)	670 nm LED	5 J/cm^2^	50 mW/cm^2^ 88 s 3 times/week for 3 weeks	Kept closed during PBM therapy, and the device was positioned 2 cm away from the eye	Improves VA and macular perimetry
AMD	Markowitz 2020 [30]	Human (46)	590 nm 660 nm 850 nm LED	None	590 nm = 5 mW/cm^2^ 660 nm = 65 mW/cm^2^ 850 nm = 8 mW/cm^2^ (250 s) (3× per week for 3–4 weeks) over 1 year	590 nm and 850 nm eyes open, 660 nm eyes closed	Improves clinical and anatomical outcomes with more robust benefits observed in subjects
AMD	Merry 2017 [2]	Human (42)	590 nm 670 nm 790 nm LED	670 nm (4–7.7 J/cm^2)^, 590 nm, 790 nm (0.1 J/cm^2^)	670 nm,50–80 mW/cm^2^ 88 s, 590 nm and 790 nm 0.6 mW for 35 s 3× per week for 3 weeks	None	Improves function and anatomical outcomes in dry AMD
DME	Shen 2020 [3]	Human (21)	670 nm Laser	2. 25 J/cm^2^ 9 J/cm^2^ 18 J/cm^2^	25, 100, or 200 mW/cm^2^ 90 s for 5 weeks	None	Reduction in CMT at all three settings at 6 months
DME	Tang 2014 [31]	Human (4)	670 nm LED	25 J/cm^2^	Twice daily for 2–9 month	Devices were held an inch away from the closed treatment eye.	Significantly reduces focal retinal thickening

VA = visual acuity, CS = contrast sensitivity, CMT = central macular thickness.

**Table 3:** Clinical studies on PBM for the treatment of retinal diseases (negative effect).

Type of Retinal Disease	First Author (Year)	Subject (No.)	λ (nm)	Dose	Irradiance Treatment Duration	Illumination Method	Result
AMD	Grewal 2020 [32]	Human (31)	670 nm LED	4.8 J/cm^2^	40 mW/cm^2^ 120 s, 12 months	looking at the red light to the front of the study eye	No effect in patients who have already progressed to intermediate AMD
DME	Kim 2022 [33]	Human (135)	670 nm LED	4.5 J/cm^2^	<50 mW/cm^2^, 90 s, twice daily for 4 months	The device is worn as a single eye patch to direct the treatment effect to the study eye	Although safe and well-tolerated, it was not found to be effective for the treatment of CI-DME in eyes with good vision
ROP	Kent 2020 [34]	Neonate < 30 weeks gestation or <1150 g (86)	670 nm LED	9 J/cm^2^	15 min daily until 34 weeks corrected age	LED was placed on the isolette 20–25 cm above the baby	This small pilot study did not show a difference in severity of ROP but may indicate an improvement in survival

CI-DME = center-involved diabetic macular edema.
